# Shiga Toxin–Producing *Escherichia coli* Outbreak in Canadian Daycare Centers

**DOI:** 10.1001/jamanetworkopen.2026.1278

**Published:** 2026-03-10

**Authors:** Mohamed Eltorki, Oluwatimilehin O. Ajayi, Jina Seok, Jianling Xie, Francesco A. Rizzuti, Byron M. Berenger, Phillip I. Tarr, Andrew T. Pavia, Kate Snedeker, Silviu Grisaru, Otto G. Vanderkooi, Linda Chui, Gillian A. M. Tarr, Gemma Vomiero, Stephen B. Freedman

**Affiliations:** 1 Section of Pediatric Emergency Medicine, Department of Pediatrics, Cumming School of Medicine, Alberta Children’s Hospital Research Institute, University of Calgary, Calgary, Alberta, Canada; 2 Department of Community Health Sciences, Cumming School of Medicine, University of Calgary, Calgary, Alberta, Canada; 3 Provincial Population & Public Health, Alberta Health Services, Calgary, Alberta, Canada; 4 Department of Pathology and Laboratory Medicine, Cumming School of Medicine, University of Calgary, Calgary, Alberta, Canada; 5 Alberta Provincial Laboratory for Public Health, Calgary, Alberta, Canada; 6 Division of Gastroenterology, Hepatology & Nutrition, Department of Pediatrics, Washington University School of Medicine in St Louis, St Louis, Missouri; 7 Division of Pediatric Infectious Diseases, Department of Pediatrics, Spencer Fox Eccles School of Medicine, University of Utah, Salt Lake City; 8 Public Health Surveillance & Informatics, Alberta Health Services, Calgary, Alberta, Canada; 9 Section of Pediatric Nephrology, Department of Pediatrics, Cumming School of Medicine, University of Calgary, Calgary, Alberta, Canada; 10 Section of Pediatric Infectious Disease, Department of Pediatrics, Cumming School of Medicine, Alberta Children’s Hospital Research Institute, University of Calgary, Calgary, Alberta, Canada; 11 Division of Environmental Health Sciences, School of Public Health, University of Minnesota, Minneapolis; 12 Section of Pediatric Hospital Medicine, Department of Pediatrics, Cumming School of Medicine, Alberta Children’s Hospital Research Institute, University of Calgary, Calgary, Alberta, Canada; 13 Section of Pediatric Emergency Medicine, Departments of Pediatrics and Emergency Medicine, Cumming School of Medicine, Alberta Children’s Hospital Research Institute, University of Calgary, Calgary, Alberta, Canada; 14 Department of Laboratory Medicine and Pathology, University of Alberta, Edmonton, Alberta, Canada

## Abstract

**Importance:**

Shiga toxin–producing *Escherichia coli* (STEC) infections have significant morbidity, with 15% to 20% of children developing hemolytic uremic syndrome (HUS), more than half of whom require kidney replacement therapy (KRT).

**Objective:**

To describe health care resource utilization, clinical outcomes, and outcomes associated with daily laboratory monitoring for thrombotic microangiopathy (TMA) in a large point-source STEC outbreak.

**Design, Setting, and Participants:**

This retrospective cohort study was conducted among a population-based cohort of individuals exposed to a contaminated food item served in 11 childcare centers in Calgary, Alberta, Canada, on August 29, 2023. Children and adults with confirmed STEC infection linked to the outbreak were included. Data were analyzed from June 9, 2025, to January 5, 2026.

**Exposure:**

*E coli* O157:H7 possessing genes encoding Shiga toxins 1 and 2.

**Main Outcomes and Measures:**

Health care utilization, symptoms and signs of infection, occurrence of HUS and KRT, and results of TMA screening.

**Results:**

There were 359 outbreak-linked confirmed STEC infections, including 285 children (median [IQR] age, 3.3 [2.3-4.2] years; 141 [62.4%] male) and 71 adults (median [IQR] age, 38.8 [31.9-46.4] years; 58 [81.7%] female) and 3 out-of-province cases; 326 primary and 33 secondary infections were identified. Of 359 infected individuals overall, 66 (18.4%) were asymptomatic. HUS occurred in 21 children (7.4%), all of whom had diarrhea (21 of 206 children with diarrhea [10.2%]). No adults developed HUS. This outbreak was linked to 508 and 395 visits by children with STEC to an emergency department and a dedicated STEC clinic, respectively; 591 visits occurred during a 7-day period, starting 6 days after exposure. Forty children were hospitalized, 9 children required KRT, and 3 children required intensive care. The most common symptoms in children and adults were diarrhea (206 children [91.2%]; 16 of 20 adults with data [80.0%]), abdominal pain (141 children [62.4%]; 11 of 20 adults with data [55.0%]), and bloody diarrhea (127 children [56.2%]; 7 of 20 adults with data [35.0%]). TMA testing had 100% (95% CI, 83.9%-100%) sensitivity, 95.7% (95% CI, 92.0%-98.0%) specificity, and a diagnostic accuracy of 96.1% (95% CI, 92.7%-98.2%) for development of HUS.

**Conclusions and Relevance:**

This cohort study reporting on a point-source STEC outbreak identified significant use of health care resources. Only 7% of children infected with STEC developed HUS, which is lower than reported in most prior outbreaks and sporadic case series. These findings highlight the importance of a coordinated public health response integrated with clinical care and suggest that proactive screening for TMA has the potential to mitigate adverse outcomes.

## Key Points

**Question:**

What were the health care resource utilization and clinical outcomes associated with a large single-source pediatric Shiga toxin–producing *Escherichia coli* (STEC) outbreak, beginning in Canadian daycare centers?

**Findings:**

In this cohort study, 326 primary and 33 secondary infections were identified among 288 children and 71 adults; among the 285 children with available outcome data, there were 508 and 395 emergency department and STEC clinic visits, respectively. Overall, 40 children were hospitalized, 21 children developed hemolytic uremic syndrome (HUS), and 9 children required dialysis; daily laboratory monitoring identified all future HUS cases.

**Meaning:**

This cohort study found that significant health care resources were used to monitor, treat, and optimize outcomes in this large single-source STEC outbreak.

## Teaser

This cohort study describes health care resource utilization, clinical outcomes, and outcomes associated with daily laboratory monitoring for thrombotic microangiopathy in a large, single-source Shiga toxin–producing *Escherichia coli* outbreak in Canadian daycare centers.

## Introduction

Shiga toxin–producing *Escherichia coli* (STEC) infection can precipitate hemolytic uremic syndrome (HUS), characterized by thrombocytopenia, microangiopathic hemolytic anemia, and acute kidney injury.^1,2,3^ HUS occurs in 15% to 20% of children infected by STEC carrying a gene encoding Shiga toxin 2 (*stx2*), which is termed *high-risk STEC*.^3^ Kidney injury leads to anuria, requiring kidney replacement therapy (KRT) in more than 50% of children with STEC-HUS.^3^ Although most infections are sporadic,^4^ outbreaks offer uniquely informative opportunities because of high ascertainment and single-strain etiology.

Alberta, Canada, has one of the highest STEC infection rates in North America.^5,6^ Accordingly, the province has developed expertise in diagnostics, public health responses, and care pathways.^7^ In 2023, the largest pediatric outbreak of high-risk STEC reported in North America occurred in Calgary among attendees, employees, and contacts of childcare centers linked to a central kitchen.^8^ This outbreak was managed through a single disease control unit within a unified health care system, with a protocolized approach to treating children infected with STEC, offering a unique opportunity to analyze timelines, resource use, clinical characteristics, laboratory testing, and outcomes among individuals who were infected.

## Methods

### Design and Objectives

This cohort study was approved by the University of Calgary’s research ethics board with a waiver of informed consent because it involved minimal-risk secondary use of health information. This study is reported in accordance with the Strengthening the Reporting of Observational Studies in Epidemiology (STROBE) reporting guideline. Data for this retrospective analysis were extracted from Alberta’s electronic health system, which integrates information from all provincial acute care centers, hospitals, and laboratories. The objectives of this study were to expand on Alberta Health Services’ Public Health Outbreak Investigation Report^8^ by quantifying health care utilization, enumerating illness characteristics, and exploring the outcomes associated with daily laboratory thrombotic microangiopathy (TMA) screening.

### Individuals With Infection

Confirmed STEC cases included individuals with test results positive for STEC using nucleic acid amplification technology or culture who were epidemiologically linked to the outbreak.^8^ Probable cases included individuals without a positive STEC test result with new-onset gastrointestinal symptoms and an epidemiological link to the source or to a confirmed case within 10 days preceding symptom onset.^8^

Six individuals with outbreak-associated infections developed symptoms on August 29, 2023; the outbreak was announced on September 4, 2023.^8^ After linking food consumption at multiple childcare centers to a shared kitchen, a public health response was initiated. The Alberta Provincial Laboratory for Public Health, Alberta Precision Laboratories distributed 5420 stool collection kits to children, staff, and others connected to affected facilities^8^; widespread screening of exposed individuals, both symptomatic and asymptomatic, began on September 5, 2023. Detailed investigations and contact tracing were conducted for all probable and confirmed cases. Primary cases were among individuals directly exposed to putatively contaminated food; secondary cases were among individuals infected through contact with an individuals with a primary case infection. Epidemiological and laboratory methods, including exposure-based linkage, probabilistic matching using identifiers, and whole-genome sequencing of STEC isolates, linked infections to the outbreak.

### Microbiology

At outbreak onset, STEC infection was identified by BD Max Enteric Bacterial polymerase chain reaction (PCR) testing of bulk stool or rectal swabs.^9^ Specimens with PCR results positive for Shiga toxin were cultured by plating onto CHROMagar STEC medium (CHROMagar) and inoculation into gram-negative broth, followed by incubation at 37 °C for 18 to 24 hours.^10,11^ Suspect colonies were subjected to laboratory-developed PCR assays to detect and differentiate *stx1* and *stx2*. To enable large-scale testing, *stx1* and *stx2* detection and differentiation were performed using laboratory-developed PCR directly on specimens with positive PCR results, with isolates undergoing molecular confirmation and whole-genome sequencing.

Serotyping and cluster analysis were performed using the whole-genome sequencing PulseNet protocol.^12^ For comparison to historical isolates, the Bactopia pipeline^13^ was used to assemble isolate genomes,^14^ IQ-TREE^15^ visualized relatedness, and JBrowse^16^ provided visualization.

### Study Definitions and Data Abstraction

We designated the date of exposure to the putatively contaminated vehicle (August 29, 2023) as outbreak day 0. TMA was present if the platelet count was less than 150 × 10^3^/µL (to convert to ×10^9^/L, multiply by 1), lactate dehydrogenase (LDH) was greater than 1000 U/L (to convert to microkatals per liter, multiply by 0.0167),^17^ or the blood smear had evidence of hemolysis (ie, >1% schistocytes).^18^ HUS was defined by the presence of hematocrit less than 30% (to convert to proportion of 1.0, multiply by 0.01), platelet count less than 150 × 10^3^/µL, and creatinine greater than the upper limit of the reference range for age.^19^ Clinical features and examination findings that were not documented were considered absent.^20^ We used explicit terminology to classify participants as ill-appearing and dehydrated.^20,21^ Significant extrarenal complications were extracted as described elsewhere.^22,23,24^

### Clinical Care

This outbreak was managed by the Calgary Zone’s Medical Officers of Health and Environmental Public Health, a unit within Alberta Health Services, Department of Provincial Population & Public Health. The Calgary Zone has a single pediatric tertiary care hospital (Alberta Children’s Hospital) and a pediatric STEC management guideline (eFigure 1 in Supplement 1) that recommends that children infected with high-risk STEC have daily blood and urine testing to identify emerging TMA and for prevention, detection, and reversal of dehydration.^25^ Hospitalization is recommended for children who appear ill, and those unable to tolerate oral fluids or with evidence of TMA. During this outbreak, children with STEC infection were directed to Alberta Children’s Hospital or a partner hospital ED where pediatric emergency care is provided 14 hours per day. Both institutions operated dedicated STEC clinics to enable daily assessments, laboratory testing, and intravenous fluid administration, if indicated.

### Statistical Analysis

Continuous and categorical variables are reported as medians and IQRs or proportions and 95% CIs, respectively. A complete-case analysis was conducted; imputation was not performed, as the missing at random assumption was unlikely to be true. Unadjusted odds ratios were estimated using Firth logistic regression due to low cell count and potential separation in the data.^26^ *P* values from multiple comparisons were adjusted using the Benjamini-Hochberg method. All statistical tests were 2-sided, and *P* < .05 was considered significant. Analyses were conducted using R software version 4.3.0 (R Project for Statistical Computing) from June 9, 2025, to January 5, 2026.

## Results

### Cases and Health Care Utilization

**Figure 1.**
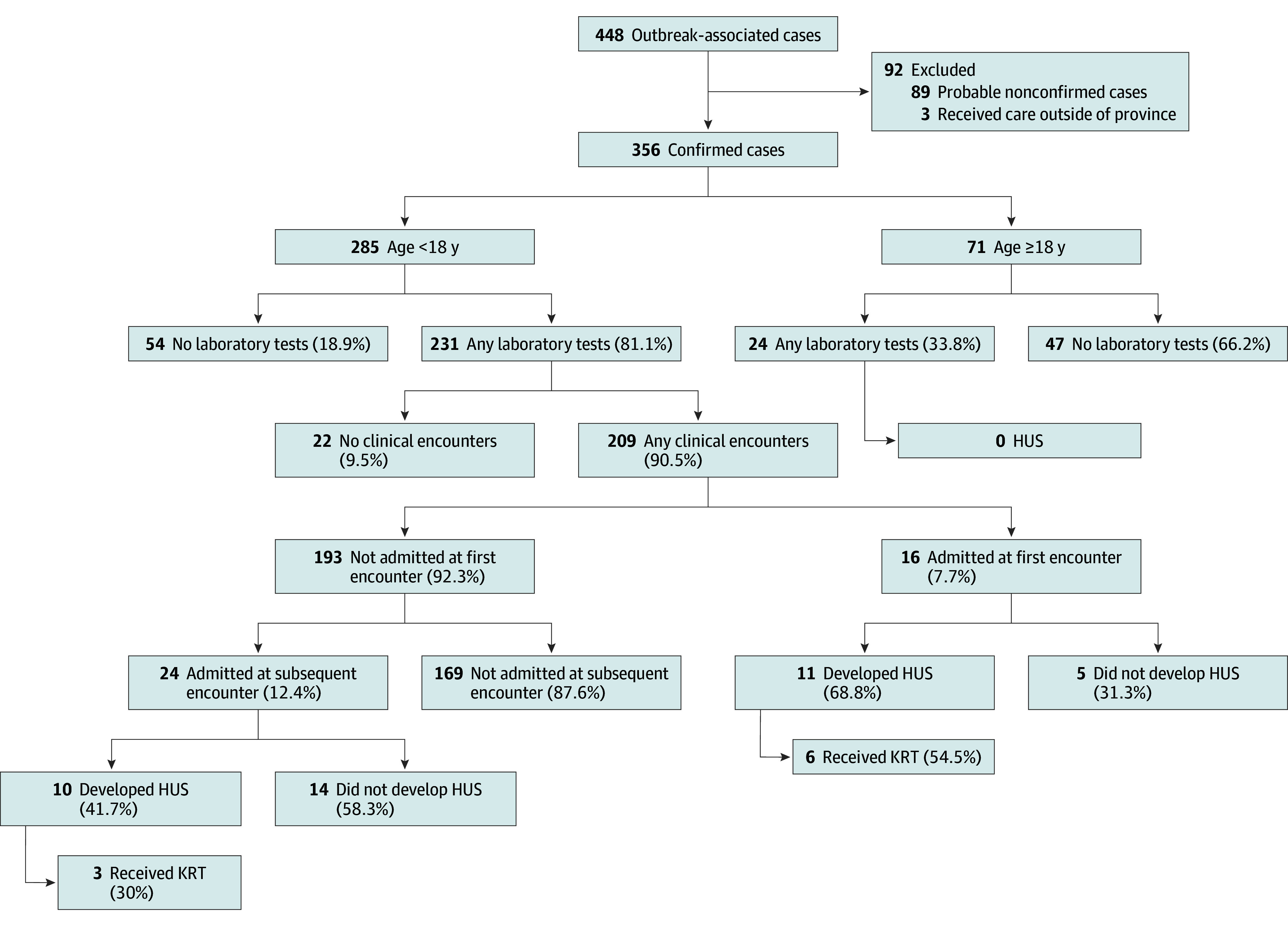
Flow Diagram Depicting Acute Care Facility Clinical Encounters, Hospitalizations, Development of the Hemolytic Uremic Syndrome (HUS) and Use of Kidney Replacement Therapy (KRT) Among Individuals With Confirmed *E coli* O157:H7 Infection

Of 359 confirmed cases of STEC, 326 (90.8%) were primary, including 271 (75.5%) among childcare attendees, 44 (12.3%) among childcare staff, 9 (2.8%) among central kitchen staff, and 2 (0.6%) among other individuals. The remaining 33 infections (9.2%) were secondary, involving household contacts, including 11 parents (3.1%), 10 siblings (2.9%), and 12 other contacts (3.3%). Of 359 confirmed infections, we report data for 356 (99.2%), 285 of whom (80.1%) were younger than 18 years (median [IQR] age, 3.3 [2.3-4.2] years; 141 [62.4%] male) and 71 were aged 18 years or older (median [IQR] age, 38.8 [31.4-46.6] years; 58 [81.7%] female); 66 individuals (18.5%) were asymptomatic; 3 individuals with STEC were diagnosed outside the province (Figure 1 and Table; eTable 2 in Supplement 1).

**Table.** Symptoms, Examination Findings, and Laboratory Characteristics of Children Evaluated at an Initial Acute Care Visit

Characteristic	Children, No. (%)^a^			*P* value^b^	OR (95% CI)^c^
	Overall (n = 226)	HUS			
		Without (n = 205)	With (n = 21)		
**Demographics**					
Age, median (IQR), y (n = 285)	3.3 (2.3-4.2)	3.3 (2.3-4.2).	3.2 (2.1-4.3)	.72	NA
Sex (n = 285)					
Female	144 (50.5)	133 (50.4)	11 (52.4)	NA	0.93 (0.38-2.23)
Male	141 (49.5)	131 (49.6)	10 (47.6)	NA	
**Clinical features**					
Diarrhea	206 (91.2)	185 (90.2)	21 (100)		4.75 (0.61-612.08)
Diarrhea duration, h	84.3 (51.2-119.8)	87.6 (54.5-122.1)	62.0 (48.2-90.5)	.20	
Diarrhea in the past 24 h,					
No.	72	60	12	NA	NA
Episodes, median (IQR), No.	9 (4-20)	9 (4-20)	8 (2-20)	.81	NA
Bloody diarrhea	127 (56.2)	106 (51.7)	21 (100)	NA	40.17 (5.42-5130.61)
Vomiting	65 (28.8)	51 (24.9)	14 (66.7)	NA	5.80 (2.33-15.57)
Vomiting duration, median (IQR), h	34.1 (15.5-71.8)	34.1 (13.6-65.0)	33.4 (22.2-88.4)	.72	NA
Vomiting in the past 24 h					
No.	22	15	7	NA	NA
Episodes, median (IQR), No.	1 (1-3)	1 (1-3)	2 (1-3)	.65	NA
Abdominal pain	141 (62.4)	121 (59.0)	20 (95.2)	NA	9.50 (2.36-86.71)
Fever	63 (27.9)	54 (26.3)	9 (42.9)	NA	2.11 (0.84-5.17)
Anuria^d^	4 (1.8)	2 (1.0)	2 (9.5)	NA	10.44 (1.54-71.16)
Tea-colored urine	1 (0.4)	0	1 (4.8)	NA	30.07 (1.55-4474.42)
Antibiotics in preceding 24 h	1 (0.4)	1 (0.5)	0	NA	3.17 (0.02-61.35)
**Physical examination^e^**					
HR>ULRR for age^23,24^	28 (12.4)	19 (9.3)	9 (42.9)	NA	7.26 (2.73-19.13)
SBP>ULRR for age^23,24^	44 (19.5)	35 (17.1)	9 (42.9)	NA	3.65 (1.43-9.12)
DBP>ULRR for age^23,24^	66 (29.2)	55 (26.8)	11 (52.4)	NA	2.97 (1.21-7.36)
Unwell appearance^f^	24 (10.6)	14 (6.8)	10 (47.6)	NA	12.06 (4.46-33.07)
Pallor	21 (9.3)	15 (7.3)	6 (28.6)	NA	5.15 (1.71-14.45)
Dehydration^g^	16 (7.1)	9 (4.4)	7 (33.3)	NA	10.70 (3.51-32.30)
Periorbital or peripheral edema	2 (0.9)	1 (0.5)	1 (4.8)	NA	9.98 (0.78-127.17)
Abdominal tenderness	11 (4.9)	8 (3.9)	3 (14.3)	NA	4.40 (1.01-15.75)
**Laboratory tests** ^h^					
White blood cells					
No. with data	127	107	20	NA	NA
Count, median (IQR), /µL	10 400 (8700-14 400)	9900 (8300-12 200)	15 800 (14 200-19 300)	<.001	NA
Neutrophils + immature white blood cells					
No. with data	125	106	19	NA	NA
Count, median (IQR), ×10^3^/µL	5.0 (3.7-8.4)	4.5 (3.6-6.4)	10.1 (8.6-14.6)	<.001	NA
Hematocrit					
No. with data	127	107	20	NA	NA
Median (IQR), %	39 (37-40)	38 (37-40)	41 (38-42)	.13	NA
Hemoglobin					
No. with data	127	107	20	NA	NA
Median (IQR), g/dL	12.9 (12.3-13.5)	12.9 (12.3-13.4)	13.4 (12.7-13.9)	.24	NA
Platelets					
No. with data	127	107	20	NA	NA
Count, median (IQR), ×10^3^/µL	324 (283-398)	319 (283-396)	348 (308-418)	.69	NA
Creatinine					
No. with data	127	107	20	NA	NA
Median (IQR), mg/dL	0.2 (0.2-0.3)	0.2 (0.2-0.3)	0.2 (0.2-0.3)	.72	NA
Blood urea nitrogen					
No. with data	122	104	18	NA	NA
Median (IQR), mg/dL	10.5 (8.5-12.6)	10.4 (8.6-12.4)	11.6 (7.7-14.4)	.65	NA
Serum sodium					
No. with data	125	106	19	NA	NA
Median (IQR), mEq/L	137 (136-138)	137 (136-139)	135 (133-136)	<.001	NA
Serum bicarbonate					
No. with data	123	104	19	NA	NA
Median (IQR), mEq/L	19 (17-21)	20 (18-21)	17 (15-18)	.001	NA
Lactate dehydrogenase					
No. with data	111	96	15	NA	NA
Median (IQR), U/L	303 (270-357)	295 (268-351)	353 (284-412)	.17	NA
C-reactive protein					
No. with data	8	4	4	NA	NA
Median (IQR), mg/L	13.9 (10.9-17.6)	16	14	.73	NA

Abbreviations: DBP, diastolic blood pressure; HR, heart rate; HUS, hemolytic uremic syndrome; NA, not applicable; OR, odds ratio; SBP, systolic blood pressure; ULRR, upper limit of reference range.

SI conversion factors: to convert blood urea nitrogen to millimoles per liter, multiply by 0.357; C-reactive protein to milligrams per liter, multiply by 10; creatinine to micromoles per liter, multiply by 88.4; hematocrit to proportion of 1.0, multiply by 0.01; hemoglobin to grams per liter, multiply by 10; lactate dehydrogenase to microkatals per liter, multiply by 0.0167; platelet count to ×10^9^/L, multiply by 1; serum bicarbonate to millimoles per liter, multiply by 1; serum sodium to millimoles per liter, multiply by 1; white blood cell count to ×10^9^/L, multiply by 0.001.

^a^ Of the 285 children with confirmed STEC infection, 59 children without an acute care visit were excluded. None of the excluded individuals developed HUS.

^b^ *P* values were adjusted using the Benjamini-Hochberg method for continuous variables. *P* value comparisons are limited to children who did and did not develop HUS.

^c^ Unadjusted ORs were estimated using Firth logistic regression because of low cell counts and potential separation.

^d^ Anuria was defined as no urine output for at least 12 hours, per parental report.^27^

^e^ No patients had jaundice or petechiae.

^f^ Unwell appearance defined as sick, toxic, shocky, decreased mental status, lethargic, unresponsive, irritable, fussy, inconsolable, not looking well, poor or decreased pulses, or similar terms.^27^

^g^ Dehydration defined as dehydrated, dry-appearing, dry mucous membranes, tented skin, sunken eyes, decreased perfusion, or similar terms.^27^

^h^ Among children who developed HUS, the median (IQR) time from the earliest blood test to meeting HUS criteria was 90.12 (70.50-98.25) hours, and laboratory testing was performed a median (IQR) of 3.0 (1.0-5.0) times.

**Figure 2.**
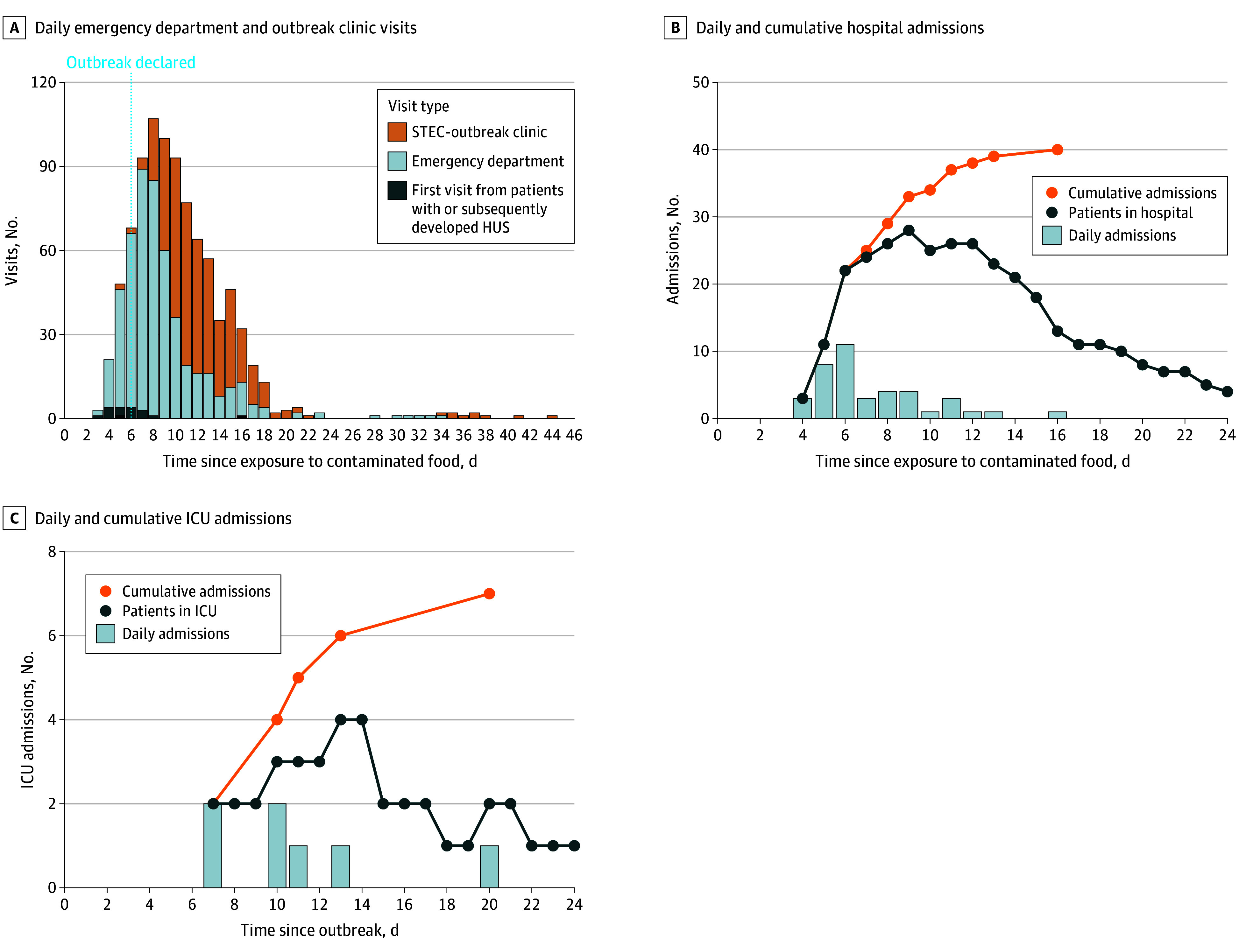
Daily Emergency Department and Outbreak Clinic Visits, With Days of First Presentation Among Children Who Developed Hemolytic Uremic Syndrome (HUS) Day 0 indicates exposure to the contaminated food item (August 29, 2023). The vertical dashed line denotes the day of the public outbreak announcement. STEC indicates Shiga toxin–producing *Escherichia coli*.

Of 285 infected children, 231 (81.0%) underwent blood testing and 214 (75.1%) were evaluated at an emergency department (ED) or urgent care center. ED visits peaked on day 7 after exposure (89 visits), and total visits (ie, acute care plus outbreak clinic) peaked on day 8 (107 visits) (Figure 2). The total number of ED and STEC clinic visits by children were 508 and 395, respectively; 591 visits occurred between September 5 and 11, inclusive. Forty children (14.0%) were hospitalized; new admissions peaked 6 days after the contaminated food item was served (6 admissions). Median (IQR) lengths of stay were 9.8 (8.6-16.7) and 2.8 (1.7-4.3) days among those with and without HUS, respectively. The maximum number of inpatients was 30, and there was a total of 346 inpatient days. Of 40 admissions, 16 (40.0%) occurred at the initial acute care evaluation. Among individuals not initially admitted, 4, 9, and 11 were admitted at the second, third or fourth, or subsequent follow-up visits, respectively. The first pediatric intensive care unit (PICU) admission occurred on day 7 postexposure, and the maximum number of concurrently hospitalized children in the PICU was 4. Three of 7 children admitted to the PICU required intensive care; the other 4 were admitted to alleviate the workload on the general pediatric unit.

### Clinical Characteristics

**Figure 3.**
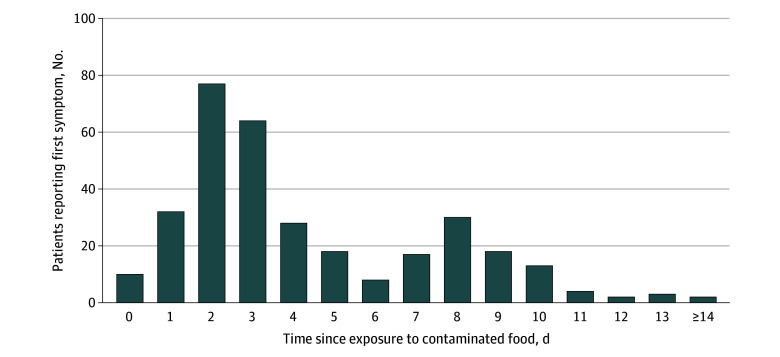
Bar Graph of Days From Exposure to Putatively Contaminated Food Item to Development of First Symptom

The incubation period of those with primary infections reveals a bimodal distribution (Figure 3). The most common symptoms at the index acute care facility visit were diarrhea (206 children [91.2%]; 16 of 20 adults with data [80.0%]), abdominal pain (141 children [62.4%]; 11 of 20 adults with data [55.0%]), and bloody diarrhea (127 children [56.2%]; 7 of 20 adults with data [35.0%]). Overall, HUS occurred in 21 children (7.4%). All children who developed HUS had diarrhea; among 206 children with diarrhea, 21 (10.2%) developed HUS. The median (IQR) time from diarrhea onset to HUS was 6.4 (5.4-7.8) days. Children who progressed to HUS were more likely to report bloody diarrhea, abdominal pain, and vomiting; they were also more likely to appear unwell, be dehydrated and tachycardic, and have higher median white blood cell counts and lower serum sodium and bicarbonate concentrations (Table).

Of 21 children who developed HUS, 9 (42.9%) received KRT. The median (IQR) duration of dialysis was 11 (8-12) days (range, 4-16 days). Extrarenal complications occurred in 3 children with HUS (eTable 1 in Supplement 1). There were no deaths.

### Microbiology

Among the 359 confirmed infections, an isolate was recovered from 339 cases, and whole-genome sequencing of these revealed a highly related strain of *E coli* O157:H7. All isolates had fewer than 10 allelic differences in core genome loci via whole-genome multilocus sequence typing, and most had 0 or 1 differences. An *E coli* O157:H7 isolate from 1 sporadic infection in Alberta in 2019 and 3 sporadic infections in 2023 also differed by fewer than 10 alleles from the outbreak strain. Outbreak isolates were within 5 core genome single-nucleotide polymorphisms of the related 2019 isolate, which belongs to a persistent lineage in Alberta.^14^ The pathogen contained *stx1* and *stx2* and belonged to cluster 3, subgroup C.^28^

The outbreak source was traced to lunch served on August 29, 2023; epidemiologic evaluation implicated beef meatloaf (adjusted risk ratio, 36.9; 95% CI, 9.2-148.1).^8^ No leftover meatloaf samples were available for testing; traceback food samples were tested at the Bureau of Microbial Hazards, Verotoxigenic *Escherichia Coli* Laboratory in Ottawa, Canada.

### Laboratory Monitoring for TMA

Among children with STEC who had blood testing performed, 30 of 231 with data (13.0%) had platelet counts less than 150 × 10^3^/µL, 21 of 229 with data (9.1%) had LDH concentrations greater than 1000 U/L, and 19 of 144 with data (17.4%) had more than 1% schistocytes on a blood smear on any individual test. Of 231 children with confirmed STEC infection, 30 (13.0%) met any of these TMA criteria at any time, among whom 21 (70.0%) developed HUS, including 21 of 30 children (70.0%) with thrombocytopenia, 19 of 21 children (90.5%) with an LDH greater than 1000 U/L, and 18 of 19 children (94.7%) with more than 1% schistocytes on a blood smear. The presence of any TMA criterion had a sensitivity of 100% (95% CI, 83.9%-100%), specificity of 95.7% (95% CI, 92.0%-98.0%), positive predictive value of 70.0% (95% CI, 50.1%-85.3%), negative predictive value of 100% (95% CI, 98.2%-100%), and diagnostic accuracy of 96.1% (95% CI, 92.7%-98.2%) for development of HUS. Only 12 children (57.1%) had clinical features (9 children with edema; 2 children with anuria; 1 child with tea-colored urine) associated with HUS at the time TMA was detected. The median (IQR) time from the earliest laboratory evidence of TMA to meeting HUS criteria was 24.3 (14.5-34.6) hours.

**Figure 4.**
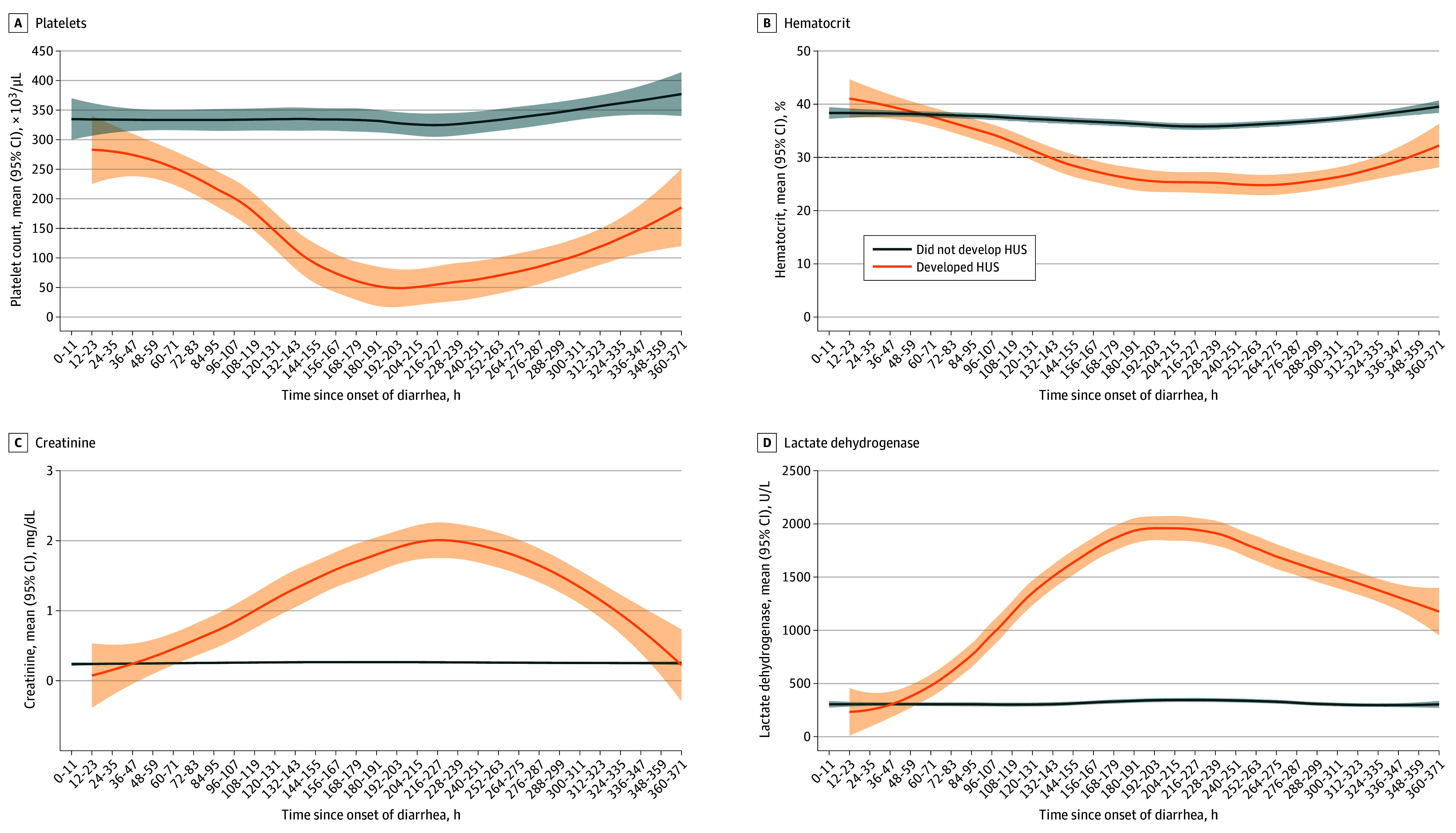
Line Graphs Showing Laboratory Values of Patients With and Without HUS The plots display the mean platelet count (A), hematocrit (B), creatinine (C), and lactate dehydrogenase (D) over time (in hours) since the onset of diarrhea, reported based on development of HUS (yes/no). The lines represent locally estimated scatterplot smoothing curves, illustrating the trends in mean values. Shaded areas indicate the 95% CIs of the estimates. The horizontal dash lines (A and B) denote the threshold for meeting specific component of the hemolytic uremic syndrome (HUS) case definition. SI conversion factors: to convert platelet count to ×10^9^/L, multiply by 1; hematocrit to proportion of 1.0, multiply by 0.01; lactate dehydrogenase to microkatals per liter, multiply by 0.0167; creatinine to micromoles per liter, multiply by 88.4.

LDH was the first parameter to diverge between patients with HUS and without HUS, becoming apparent 60 hours after diarrhea onset (Figure 4). Approximately 70% of children who developed thrombocytopenia (ie, platelets <150 × 10^3^/µL) crossed this threshold by 132 hours. Hemoglobin and hematocrit concentrations were initially higher among children who developed HUS compared with those who did not, with the curve for those who developed HUS crossing the 30% value for hematocrit and 10 g/dL value for hemoglobin (to grams per liter multiply by 10), approximately 130 hours following symptom onset (eFigure 2 in Supplement 1). Creatinine levels increased slowly, with the central tendency only exceeding 1.0 mg/dL (to convert to micromoles per liter, multiply by 88.4) 110 hours after diarrhea onset.

## Discussion

In this cohort study, we describe health care resource utilization, clinical outcomes, and the outcomes associated with daily laboratory monitoring for TMA in a large point-source STEC outbreak. STEC outbreaks provide unique opportunities to study these complex infections because of high case ascertainment and shared pathogen exposure. This outbreak provides additional insight, given the young age of the affected population, the point source nature of the exposure, the comprehensive public health investigation, and the presence of a single laboratory and health system with a standardized approach to diagnosis and management.

This outbreak is notable for the low rate of HUS, despite the infected population being at high risk for the development of this complication.^27^ Shiga toxin subtype determines HUS risk, with isolates testing positive for *stx1* and *stx2* having a somewhat lower likelihood of this outcome than isolates testing negative for *stx1* and *stx2*.^29^ In the 1993 Washington state outbreak, the HUS rate among patients younger than age 16 years was 13.3%, and the mortality rate among those who developed HUS was 8.1%.^30^ In the 1996 outbreak in Sakai, Japan, the HUS rate was 15.5% among children aged 6 to 7 years,^31^ and the fatality rate was 2.5% among children with HUS.^32^ In Walkerton, Ontario, in 2000, among 163 children infected with *E coli* O157, 14.7% developed HUS, 5.7% required dialysis, and 0.6% died.^33^

Nonoutbreak studies of *stx1*- and *stx2*-positive *E coli* O157 infections have reported worse outcomes than we report in this study, particularly among children younger than 5 years. In a report of 85 children infected with *E coli* O157, while the overall rate of HUS was 11%, children younger than 5 years had an increased risk of developing HUS (adjusted odds ratio, 10.9).^34^ Other reports describe a 15% to 20% HUS rate among children younger than 5 years infected with O157.^34,35^ In the outbreak examined here, the use of KRT among individuals who developed HUS was also lower than is typically reported (50%-65%).^36,37,38,39^

In observational studies such as ours, outcomes cannot be attributed with confidence to the presence or absence of any intervention. As previously noted, differences in pathogen virulence and the age of children with STEC are unlikely contributors to our low complication rate, as the cohort was uniformly young and infected by strains with toxin profiles similar to comparator outbreaks. Although inoculum size may influence outcomes, data on individual exposure levels were unavailable, and, to our knowledge, there are no studies associating inoculum with outcomes in individuals with STEC infection.

We believe the systematized approach to monitoring and managing patients with STEC infection may have played an important role. In this outbreak, all exposed individuals were tested, and all individuals with test results positive for STEC were connected to follow-up care, with most treated in accordance with a clinical care pathway, including the performance of daily blood tests and intravenous fluid administration as needed. This facilitated detection of TMA and led to hospitalization of numerous children who had evidence of TMA or HUS during follow-up visits. Importantly, laboratory evidence of TMA was detected a median of 24 hours before HUS criteria were met. We believe this window, which enabled the prevention or reversal of dehydration and enhanced monitoring and initiation of treatment, as appropriate, played a role in minimizing severe adverse outcomes.^40^

Our description of the evolution of TMA, with laboratory parameters initially within reference ranges, highlights the need for ongoing daily laboratory monitoring. Furthermore, we report that TMA developed in children who did not progress to HUS (ie, did not experience elevated serum creatinine concentration), suggesting that some degree of acute kidney injury likely occurs in the absence of development of HUS.^41^ The importance of close monitoring of laboratory parameters is magnified by the paucity of clinical signs that would prompt medical evaluation in our cohort of children who developed HUS. Efforts by Alberta’s public health system for comprehensive contact tracing, case identification and screening, and public announcement of the outbreak may have contributed to improved outcomes through early pathogen identification, early presentation to care, prevention of dehydration, and timely recognition of evolving HUS. The public health system response also likely led to the identification of asymptomatic children and others with mild disease; such children do not routinely seek medical attention or have stool tested and are less likely to develop HUS.^42^ Swift public health control measures provided individuals with infection and their families information in a timely manner, thereby reducing secondary spread.

The impact on the health care system and resources associated with this single-exposure STEC outbreak included an early peak in ambulatory visits followed by a delayed surge in hospitalizations. This aligns with the Sakai outbreak, where hospital visits peaked on days 2 to 3 and HUS presentations occurred 6 to 9 days after the index case presented.^32^ During the O157 outbreak in Washington state in 1993, the local tertiary pediatric institution was presented as a resource for families of children with signs of infection, after which a 17% increase in patient visits and 103% increase in the number of children with gastrointestinal illness occurred over a 2-month period.^43^ In contrast, the Calgary outbreak was managed with a novel hub-and-spoke model, with the tertiary care center providing care to individuals with HUS while STEC outbreak clinics provided ambulatory services.

This outbreak enabled the precise examination of the incubation period of *E coli* O157:H7. Interestingly, the bimodal nature of the incubation period may reflect the fact that some children meeting the primary case definition (ie, exposure to infected food item) may have been secondarily infected. Our data also highlight the indirect impact on caregivers and childcare workers. Most adult secondary cases were parents, most of whom were women, and more than 90% of childcare facility staff who were infected were female.^44^ These patterns reflect broader gendered caregiving dynamics documented across health care systems.^45^ Other public health emergencies, including COVID-19, have similarly shown that female caregivers face higher rates of workforce disruption, mental health strain, and reduced economic stability.^46,47^

### Limitations

This study has limitations. The retrospective design and lack of comprehensive clinical and laboratory data on all individuals with STEC infection preclude ruling out subclinical evidence of TMA. We did not have access to primary care practitioner visits and volume of intravenous fluids administered at each visit and thus are unable to comment on their impacts. In addition, although the outbreak investigation by Calgary Zone’s Medical Officers of Health and Environmental Public Health unit classified individual cases as primary or secondary, due to provincial privacy legislation, these data could not be shared with our team at a patient level; as such, we cannot confirm that the bimodal distribution of time to first symptom onset indeed reflects primary vs secondary cases. The generalizability of our outcomes may be limited by the unique structure of Alberta’s integrated health care system, which facilitated centralized outbreak management and consistent laboratory follow-up.

## Conclusions

This cohort study describing a large pediatric outbreak of STEC underscores the importance of coordinated public health responses and standardized protocols. Early identification of TMA and evolving HUS can enable the timely identification of children at risk for complications and has the potential to reduce adverse outcomes. The information on timing and strain on health care resources that we present can inform future outbreak mitigation, preparedness, and response strategies.

## Notes

Supplement 1. eTable 1. Complications and invasive procedures occurring within 30 days of a positive Shiga toxin-producing *E. coli* test eFigure 1. Conservative fluid management clinical care pathway implemented in Calgary during the daycare outbreak eFigure 2. Laboratory values over time eTable 2. Symptoms, examination findings, and laboratory characteristics of adults with confirmed STEC infection at the initial acute care visit

Supplement 2. Data Sharing Statement
