# Error in Figure 2

**DOI:** 10.1001/jamanetworkopen.2026.10784

**Published:** 2026-04-13

In the Original Investigation titled “Shiga Toxin–Producing *Escherichia coli* Outbreak in Canadian Daycare Centers,”^1^ published March 10, 2026, some hospital admissions were counted twice in the cumulative incidence data in Figure 2B. This article has been corrected.^1^
