# Supplementary material for: Shiga Toxin–Producing Escherichia coli Outbreak in Canadian Daycare Centers
Source: JAMA Netw Open. 2026 Mar 10;9(3):e261278. doi: 10.1001/jamanetworkopen.2026.1278 (PMC12976786; doi:10.1001/jamanetworkopen.2026.1278)
Supplement: Supplement 1. — eTable 1. Complications and invasive procedures occurring within 30 days of a positive Shiga toxin-producing E. coli test eFigure 1. Conservative fluid management clinical care pathway implemented in Calgary during the daycare outbreak eFigure 2. Laboratory values over time eTable 2. Symptoms, examination findings, and laboratory characteristics of adults with confirmed STEC infection at the initial acute care visit [file jamanetwopen-e261278-s001.pdf]

Supplemental Online Content

Eltorki M, Ajayi OO, Seok J, et al. Shiga toxin–producing *Escherichia coli* outbreak in Canadian daycare centers. *JAMA Netw Open*. 2026;9(3):e261278. doi:10.1001/jamanetworkopen.2026.1278

eTable 1. Complications and invasive procedures occurring within 30 days of a positive Shiga toxin-producing *E. coli* test
eFigure 1. Conservative fluid management clinical care pathway implemented in Calgary during the daycare outbreak
eFigure 2. Laboratory values over time
eTable 2. Symptoms, examination findings, and laboratory characteristics of adults with confirmed STEC infection at the initial acute care visit

This supplemental material has been provided by the authors to give readers additional information about their work.

© 2026 Eltorki M et al. *JAMA Network Open*.

Supplemental Table S1: Complications and invasive procedures occurring within 30 days of a positive Shiga toxin-producing *E. coli* test.†

	All Children (N=285)	Without HUS† (N=264)	With HUS (N=21)
Renal failure requiring kidney replacement therapy, yes (%)	9 (3.2%)	0 (0%)	9 (42.9%)
Any PICU Admission	7 (2.5%)	0 (0%)	7 (33.3%)
**Complications**			
Neurologic Complication	2 (0.7%)	0 (0%)	2 (9.5%)
• Coma	1 (0.4%)	0 (0%)	1 (4.8%)
• Seizure requiring anti-convulsant therapy	2 (0.7%)	0 (0%)	2 (9.5%)
Respiratory Complication	3 (1.1%)	0 (0%)	3 (14.3%)
• Pleural effusion requiring drain	1 (0.4%)	0 (0%)	1 (4.8%)
• Respiratory failure	3 (1.1%)	0 (0%)	3 (14.3%)
Infectious Complication	1 (0.4%)	0 (0%)	1 (4.8%)
• Septic shock/peritonitis	1 (0.4%)	0 (0%)	1 (4.8%)
Gastrointestinal Complication	2 (0.7%)	1 (0.4%)	1 (4.8%)
• Bowel perforation requiring resection	1 (0.4%)	0 (0%)	1 (4.8%)
• Intussusception	1 (0.4%)	1 (0.4%)	0 (0%)
**Invasive Procedures**			
Any Invasive Medical Procedures	17 (6%)	1 (0.4%)*	16 (76.2%)
Central Line Insertion	16 (5.6%)	0 (0.4%)	15 (71.4%)
Peritoneal Dialysis Catheter	9 (3.2%)	0 (0%)	9 (42.9%)
Mechanical Ventilation for Respiratory Support	3 (1.1)	0 (0)	3 (14.3%)

PICU, Pediatric Intensive Care Unit; HUS, Hemolytic Uremic Syndrome.
†Some children experienced multiple complications.
¶Any Complication or Invasive Procedure includes neurologic, respiratory, infectious, gastrointestinal complications, or invasive medical procedures.
*1 Child without HUS had a protein losing enteropathy, had a peripherally inserted central venous catheter performed under general anesthesia to enable total parenteral nutrition administration. The lowest platelet count was 99 x 10^3/µL, hemoglobin of 7.2 g/dL (a packed red blood cell transfusion was provided), and maximal creatinine value of 0.4 mg/dL.

© 2026 Eltorki M et al. *JAMA Network Open*.

Supplemental Figure S1: Conservative Fluid Management Clinical Care Pathway implemented in Calgary during the Daycare Outbreak

Children potentially eligible for this pathway include all of those with evidence of STEC infection (i.e. detection of Shiga toxin, Shiga toxin-producing E. coli, or early HUS) or clinical presentation highly suspicious for STEC infection

The following flow diagram identifies children likely to be infected by a **high-risk** STEC and who should be started on the conservative pathway

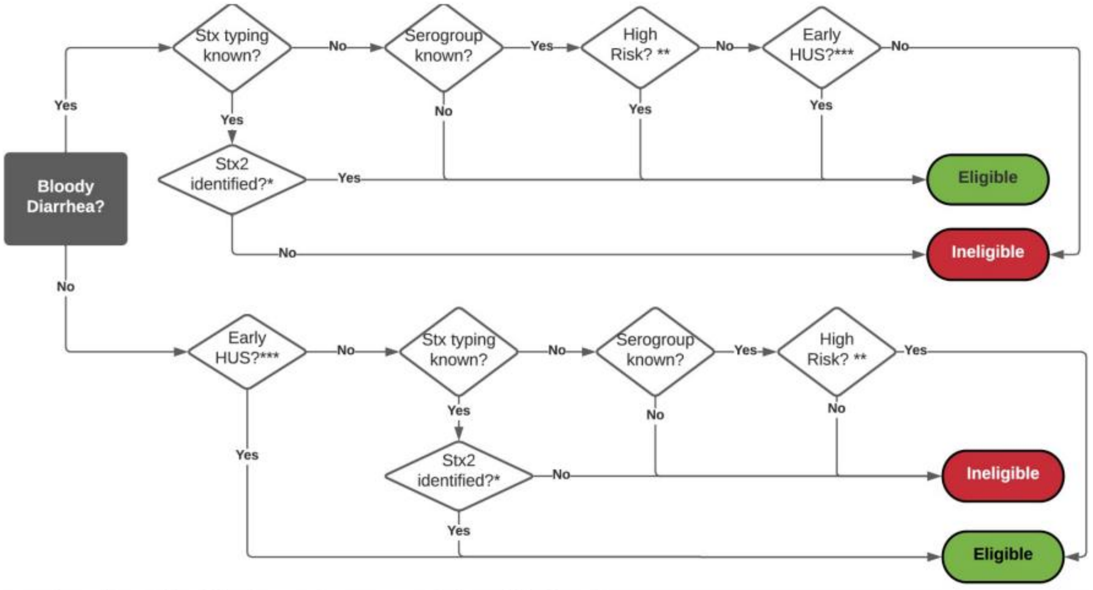

*Isolated Stx1 identification confers extremely low risk of HUS
**The following serogroups are high risk: O103, O104, O111, O113, O121, O145, and O157
***Meets standard HUS criteria [anemia, thrombocytopenia and elevated serum creatinine, but absolute value is ≤ 2.O mg/dL (177 μmol/L)]

<u>Admission Recommended if:</u>
ANY of the following (see Admission Protocol on next page):

1. Ill-appearing
2. Unable to tolerate PO fluids
3. Any labs concerning for HUS:
   i. Anemia
   ii. Thrombocytopenia
   iii. Elevated creatinine
   iv. LDH > 2xULN

<u>Outpatient Management Recommendations</u>

1. Encourage ORAL fluid intake
2. Blood tests approximately Q24 (CBC, electrolytes, creatinine, urea, LDH)
3. Seek ED care if any of the following:
   i. New bleeding, bruising, petechial rash
   ii. Severe abdominal pain
   iii. Unusual/severe headache
   iv. Tea-colored urine
   v. No urine output for >12 hours vi.

<u>Follow-up Plan:</u>

1. Follow-up should include laboratory and hydration evaluation every 24 hours until termination criteria are met
2. Seek ED care if concerning laboratory or clinical findings

© 2026 Eltorki M et al. *JAMA Network Open*.

Admission Protocol (if deemed required)

Management

- Reverse dehydration
- Target Euvolemia taking into consideration ongoing losses (e.g., vomiting, diarrhea, third spacing)
- Limit weight gain to <5% above baseline weight

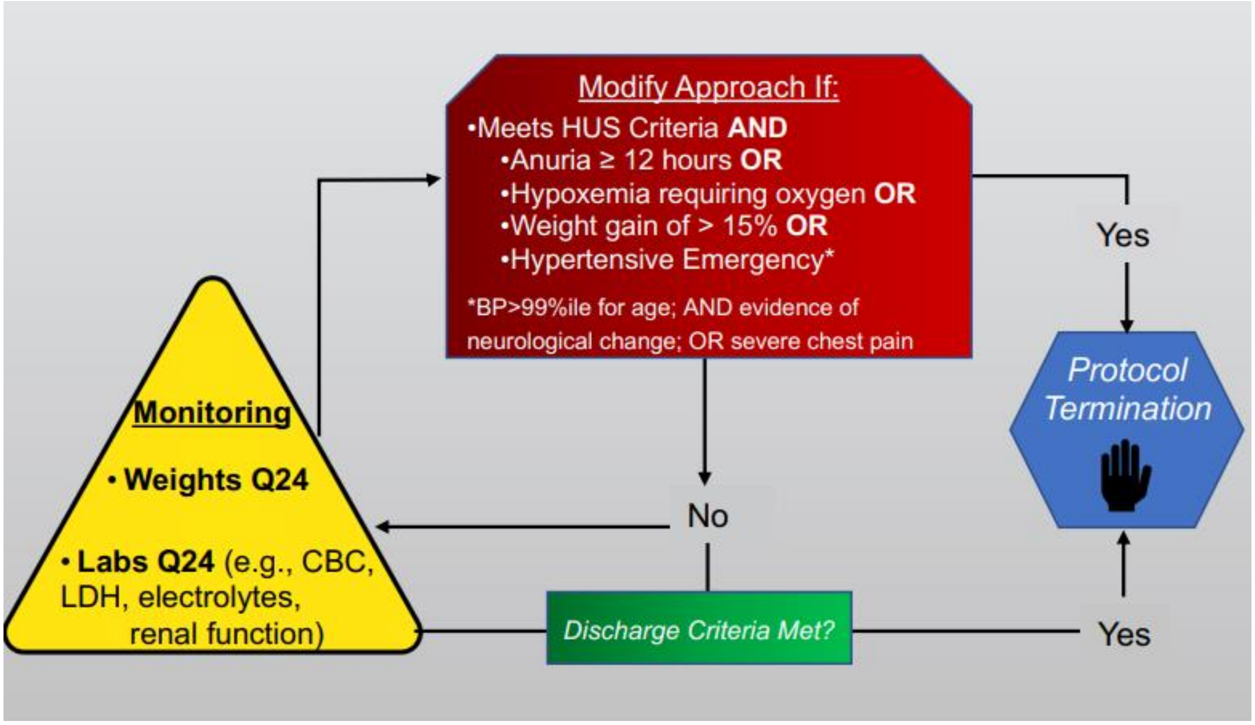

General Recommendations

- Administer isotonic fluids (ideally balanced crystalloid) at a rate of 1x maintenance as calculated using the Holliday-Segar formula
- Intravenous fluid solutions containing < 130 mEq/L sodium should be avoided
- Consider nephrology consultation for all children

Discharge Criteria

STEC Resolution: Platelet count is >50 x 109/L (in absence of transfusion) AND Platelets have increased by ≥5%* (in absence of transfusion) since preceding test AND Absence of diarrhea (loose or watery stool) x 24 hours AND ≥5 days since the onset of diarrhea

*If >10 days since the onset of diarrhea, up to a 5% decrease in platelet count since preceding test is acceptable.

© 2026 Eltorki M et al. *JAMA Network Open*.

Supplemental Figure S2: Laboratory values over time. The plots display hemoglobin over time (in hours) since the onset of diarrhea, reported based on development of HUS (Yes/No). The lines represent LOESS (Locally Estimated Scatterplot Smoothing) curves, illustrating the trends in mean values. Shaded areas indicate the 95% confidence intervals of the LOESS estimates. The numbers alongside the lines represent the number of individuals included in the mean calculation at each time point.

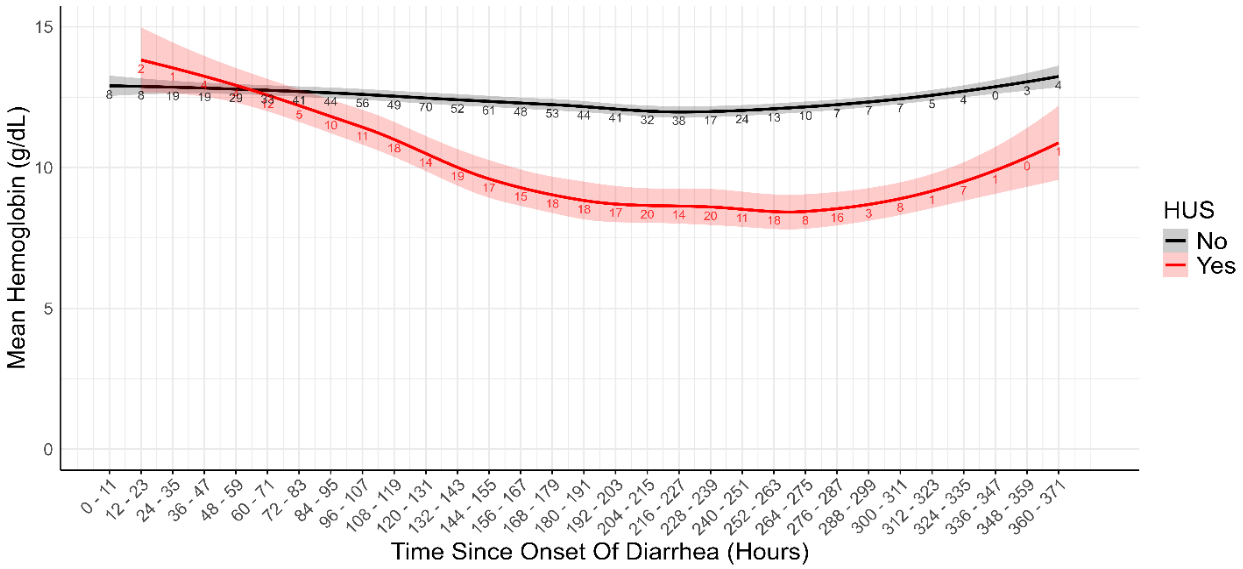

Supplemental Table S2: Symptoms, examination findings, and laboratory characteristics of adults with confirmed STEC infection at the initial acute care visit

**Characteristic**	**Adults[§]**<br>**N=20**<br>**Overall N (%) or Median (IQR)[¶]**
Age, years	38.8 (31.9, 46.4)
Male sex	13 (65.0)
Diarrhea	16 (80.0)
Diarrhea duration, hours	88.6 (64.7, 147.9) (n=16)
Diarrhea episodes past 24 hours	10 (6, 10)<br>(n=6)
Bloody diarrhea	7 (35.0)

© 2026 Eltorki M et al. *JAMA Network Open*.

Vomiting	6 (30.0)
Vomiting duration, hours	65.6 (42.2, 87.4) (n=6)
Vomiting episodes past 24 hours	4<br>(n=2)
Abdominal pain	11 (55.0)
Fever	3 (15.0)
Anuria**	0 (0.0)
Tea-colored urine	0 (0.0)
Antibiotics in preceding 24 hours	0 (0.0)
HR >ULN for age[53,54]	2 (10.0)
SBP >ULN for age[53,54]	10 (50.0)
DBP >ULN for age[53,54]	8 (40.0)
Unwell appearance#	3 (15.0)
Pallor	3 (15.0)
Dehydration‡	3 (15.0)
Periorbital or peripheral Edema	0 (0)
Abdominal Tenderness	7 (45.0)
White blood cells x 10^3/μL	10.6 (7.3, 12.3)<br>(n=9)
Neutrophils plus Immature white blood cells x 10^3/μL	8.2 (6.5, 9.2)<br>(n=9)
Hematocrit (%)	44 (43, 45)<br>(n=9)
Hemoglobin, g/dL	15.0 (14.9, 15.4)<br>(n=9)
Platelets count, x 10^3/μL	279 (234, 298)<br>(n=9)
Creatinine, mg/dL	0.6 (0.6, 0.7)<br>(n=10)
Blood urea nitrogen, mg/dL	9.4 (6.9, 10.9)<br>(n=6)
Serum sodium, mEq/L	138 (137, 139)<br>(n=9)
Serum bicarbonate, mEq/L	24 (22, 25)

© 2026 Eltorki M et al. *JAMA Network Open*.

	(n=9)
Lactate dehydrogenase, U/L	177 (175, 187)<br>(n=5)
C-reactive protein, mg/L	5.5<br>(n=1)

Hemolytic Uremic Syndrome, HUS; Heart Rate, HR; Systolic Blood Pressure, SBP; Diastolic Blood Pressure, DBP; Upper Limit of Normal, ULN. §No adults had HUS ¶Values are presented as N (%), or median (interquartile range [IQR]); IQR is only reported when a minimum of 5 observations is available. ***Of 71 adults with confirmed STEC infection, 51 adults without an acute care visit were excluded, leaving 20 adults; none of the excluded individuals developed HUS.** **Anuria=no urine output ≥12 hours, per parental report[26] #Unwell appearance=sick, toxic, shocky, decreased mental status, lethargic, unresponsive, irritable, fussy, inconsolable, not looking well, poor or decreased pulses, decreased pulses, or other similar terms.[26] ‡Dehydration=Dehydrated, dry-appearing, dry mucous membranes, tented skin, sunken eyes, decreased perfusion, or other similar terms.[26] §No patients had jaundice or petechia.

© 2026 Eltorki M et al. *JAMA Network Open*.
