# Supplementary material for: Shiga Toxin–Producing Escherichia coli Outbreak in Canadian Daycare Centers
Source: JAMA Netw Open. 2026 Mar 10;9(3):e261278. doi: 10.1001/jamanetworkopen.2026.1278 (PMC12976786; doi:10.1001/jamanetworkopen.2026.1278)
Supplement: Supplement 2. — Data Sharing Statement [file jamanetwopen-e261278-s002.pdf]

Data Sharing Statement

Eltorki. Shiga Toxin–Producing Escherichia coli Outbreak in Canadian Daycare Centers. *JAMA Netw Open*. Published March 10, 2026. doi:10.1001/jamanetworkopen.2026.1278

Data
Data available: No

Additional Information
Explanation for why data not available: Alberta Health Services is the custodian of the data. Every request for data will be evaluated and data will be made available if the custodian has no privacy concerns.
